# CAR-T cell manufacturing landscape—Lessons from the past decade and considerations for early clinical development

**DOI:** 10.1016/j.omtm.2024.101250

**Published:** 2024-04-16

**Authors:** Juliana Dias, John Garcia, Giulia Agliardi, Claire Roddie

**Affiliations:** 1 Centre for Cell, Gene and Tissue Therapeutics, Royal Free Hospital NHS Foundation Trust, London NW3 2QG, UK; 2 Research Department of Haematology, Cancer Institute, University College London, London WC1E 6DD, UK

**Keywords:** chimeric antigen receptors, immunotherapy, manufacturing, automation, quality control

## Abstract

CAR-T cell therapies have consolidated their position over the last decade as an effective alternative to conventional chemotherapies for the treatment of a number of hematological malignancies. With an exponential increase in the number of commercial therapies and hundreds of phase 1 trials exploring CAR-T cell efficacy in different settings (including autoimmunity and solid tumors), demand for manufacturing capabilities in recent years has considerably increased. In this review, we explore the current landscape of CAR-T cell manufacturing and discuss some of the challenges limiting production capacity worldwide. We describe the latest technical developments in GMP production platform design to facilitate the delivery of a range of increasingly complex CAR-T cell products, and the challenges associated with translation of new scientific developments into clinical products for patients. We explore all aspects of the manufacturing process, namely early development, manufacturing technology, quality control, and the requirements for industrial scaling. Finally, we discuss the challenges faced as a small academic team, responsible for the delivery of a high number of innovative products to patients. We describe our experience in the setup of an effective bench-to-clinic pipeline, with a streamlined workflow, for implementation of a diverse portfolio of phase 1 trials.

## Graphical abstract

**Figure:**
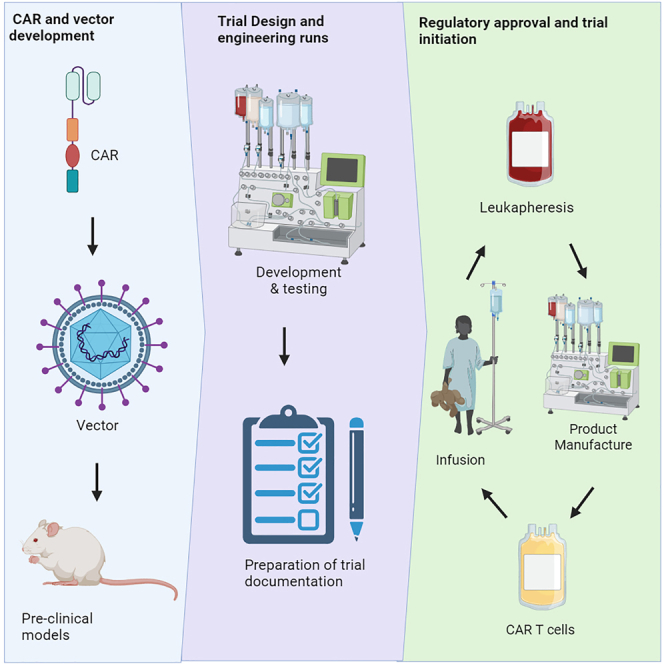

## Teaser

Dias and colleagues assess in this review the current CAR-T cell manufacturing landscape, considering challenges that will need to be addressed to facilitate translation of the next generation of these advanced therapies into clinic from an academic perspective.

## Introduction

Chimeric antigen receptor (CAR) T cell therapy has emerged as a ground-breaking immunotherapeutic approach, with remarkable efficacy especially in refractory hematologic malignancies. Data indicate that CAR-T cell manufacturing processes can impact clinical outcomes^1^^,^^2^ and manufacturing science has evolved substantially over the past decade.

As the demand for CAR-T cell therapies grows, with expansion of applicability toward new indications and to attend a yet unmet need in developing countries,^3^ it becomes essential to implement good manufacturing practice (GMP) strategies that are flexible and robust, with streamlined workflows and optimized production efficiency. In parallel, development of new and more complex products is associated with a heavy demand for assay development and quality control strategies to ensure batch-to-batch consistency and safety.

The enormous innovation in CAR-T cell research and development far surpasses current capacity for clinical manufacture worldwide. Production requires the use of specialized cleanroom facilities and, more importantly, skilled workforce. Gap analysis by work groups both in the UK and the US highlight the shortage of qualified manufacturing staff as one of the main challenges to address in the Cell and Gene Therapy field.^4^^,^^5^ Importantly, a recent review of CAR-T manufacturing processes estimates that over 200 labor hours are required per batch/lot, inclusive of manufacturing, quality assurance (QA), quality control (QC), logistics and supply chain management. This represents 71% of the total costs associated with batch production, with as much as 48% of costs deriving from the manufacturing labor alone.^6^

This comprehensive review of the CAR-T cell manufacturing landscape seeks to provide critical insights into advancements that have shaped the field, with a focus on novel technical and methodological developments that have the potential to increase product quality and patient access.

## Manufacturing

Despite the rapid advance of technology, the general workflow for CAR-T cell manufacture has remained consistent over the years, even from the first clinical trials.^7^ Briefly, autologous peripheral blood mononuclear cells (PBMCs) are procured via unstimulated leukapheresis, are enriched for T cells using magnetic beads or density-based methods, are then activated and transduced *in vitro* with a lentiviral or retroviral vector encoding the CAR cassette, and expanded until therapeutic CAR-T cell doses are achieved. This general protocol has been employed with minor modifications over the last decade for hundreds of clinical trials, as well as for commercial production.

**Figure 1:**
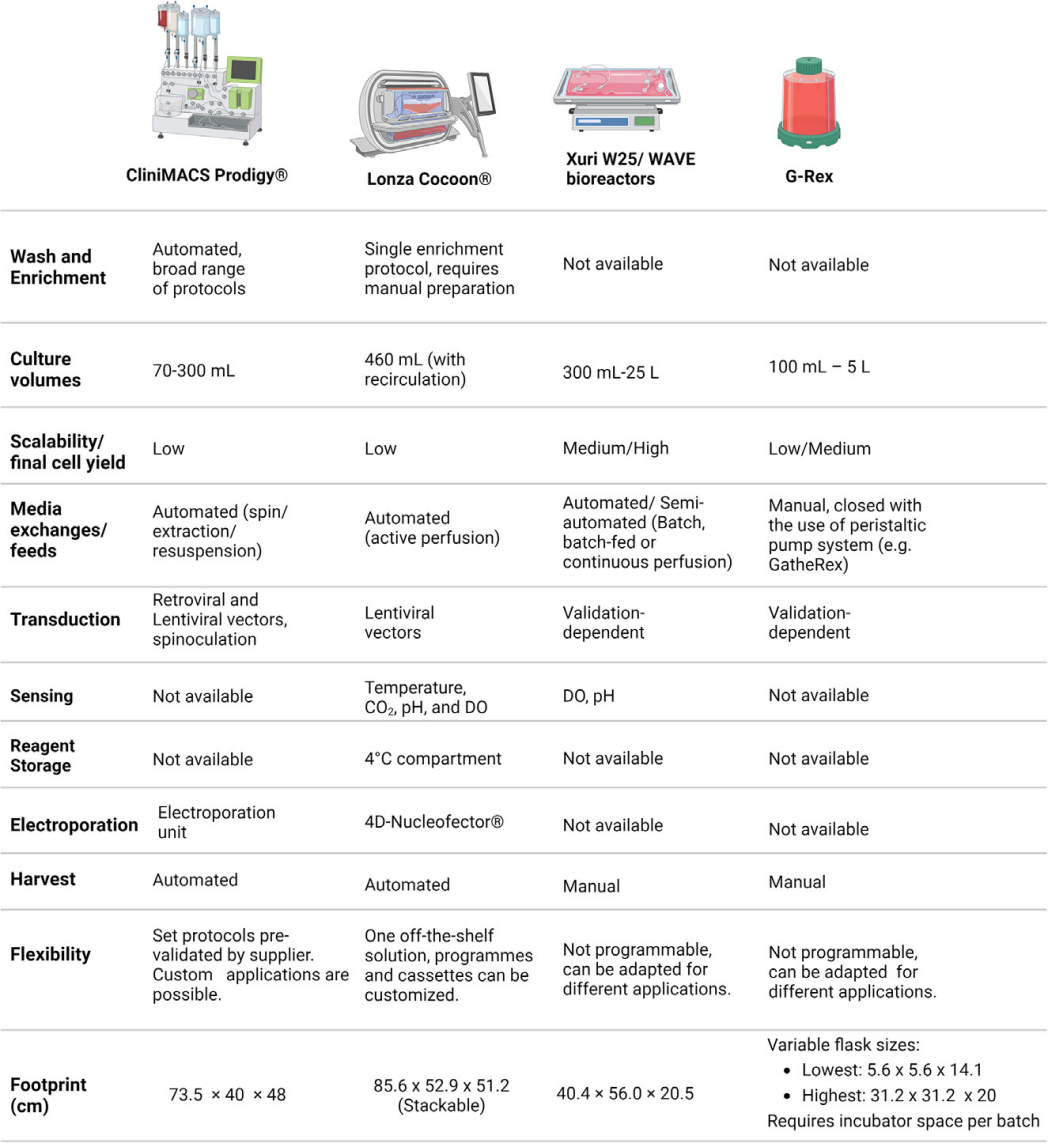
Overview of the different CAR-T production platforms available in the market These devices vary widely on level of automation, capacity, and integration with additional processing modules. The footprint has been evaluated taking into account requirements for additional pieces of equipment. Note that volumes and numbers for the CliniMACS Prodigy consider the standard application, but a large-scale protocol has recently been released by the supplier and validated.^134^

**Figure 2:**
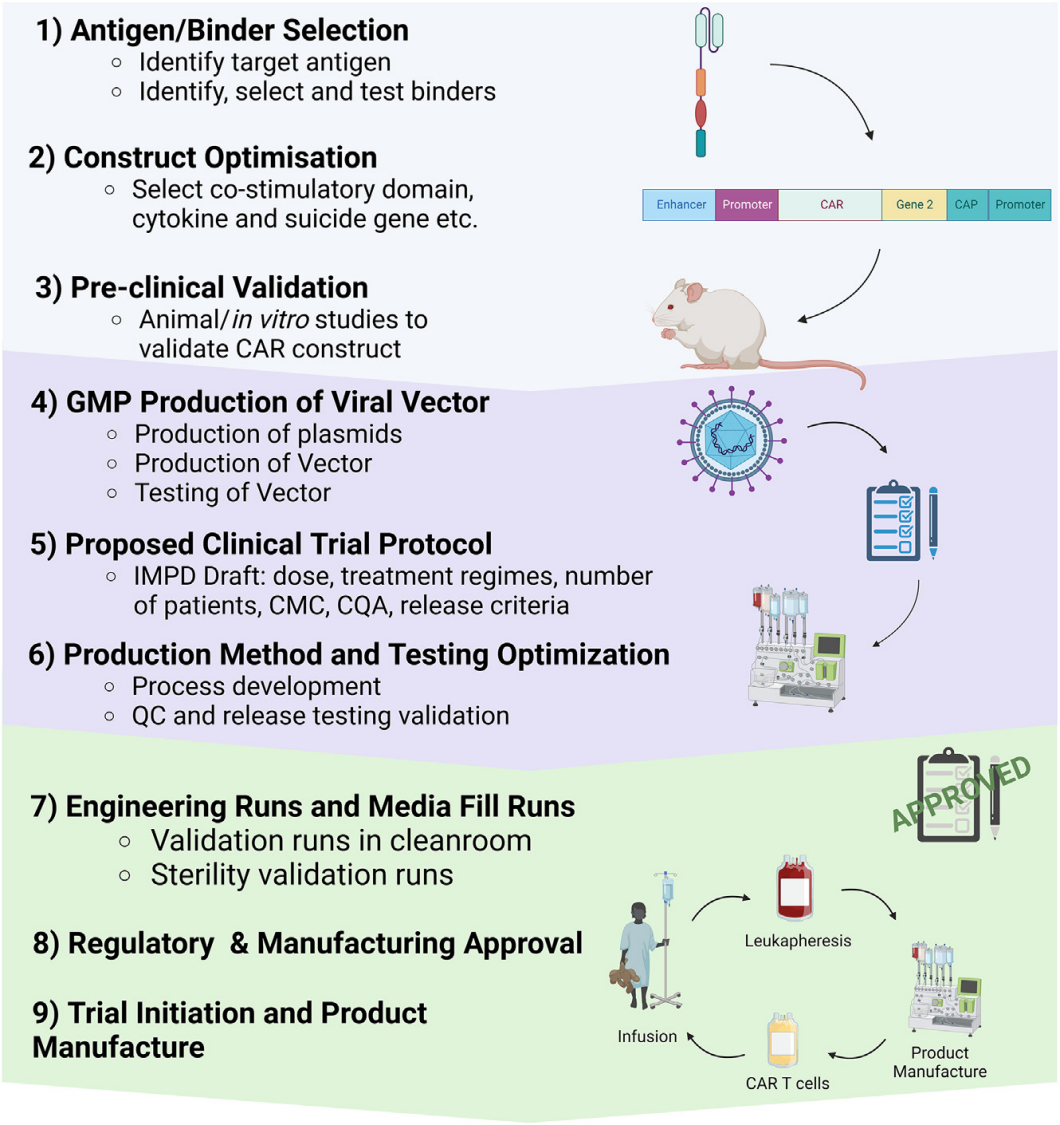
Bench-to-bedside workflow used in the UCL program for translation and delivery of new phase 1 CAR-T cell studies New target antigens and specific binders are identified in the research lab and CAR constructs optimized for the desired application (e.g., co-stimulatory domains, cytokine secretion, suicide or marker genes, etc.). The chosen CARs are validated *in vitro* and with the use of animal models and, if applicable, genome-editing strategies are also validated. A viral vector batch is then manufactured under GMP compliance. At this stage, it is expected that a proposed trial protocol is available, indicating the treatment regimens and required doses, as this will help defining the critical quality attributes of the drug product. Based on this information, the proposed manufacturing protocol is adjusted, a testing plan is devised, and testing methods optimized, before scale-up engineering runs are completed to show that products can be manufactured with the desired characteristics. All data on literature review and trial rationale, ethical considerations, risk-benefit analysis, pre-clinical validation data, detailed manufacturing methods and compliance, drug product characteristics, and proposed protocol are submitted for regulatory review by the MHRA in the UK. Once a CTA is granted, internal protocols are followed to complete the required validations and obtain the relevant approvals. The team is then trained on the pre-approved manufacturing and testing methods and products are manufacture in accordance with issued SOPs. Each batch manufactured undergoes detailed review by the quality team and the Qualified Person (QP) prior to release and results are constantly monitored to continuously improve processes. CAR, chimeric antigen receptors; CMC, chemistry, manufacturing, and control; CQA, critical quality attributes; QC, quality control; cGMP, current good manufacturing practice; MHRA, Medicines and Healthcare Products Regulatory Agency; CTA, clinical trial authorization.

Although methods vary between laboratories, technical and methodological developments in CAR-T cell manufacturing have in general focused on four main aspects: (1) a shift from manual methods to closed semi-automated systems (Figure 1), (2) improvement of product phenotype, (3) development of rapid, no-expansion protocols, and (4) the emerging use of genome-editing tools.

### Automation

#### Starting materials and enrichment

Current CAR-T manufacturing protocols can use a wide range of processing strategies to achieve starting materials with the desired characteristics. Most manufacturing protocols use autologous PBMCs, procured via unstimulated leukapheresis using standard outpatient protocols. This usually permits collection of high T cell yields toward targets set by manufacturers (often in excess of 1 billion T cells), even in patients with low peripheral blood lymphocyte counts, but manufacturing failures still occur due to poor T cell quality and yields.^8^

More recently, improved T cell activation and expansion technologies have permitted a significant reduction in starting T cell number requirements such that a 25- to 100-fold expansion can often be achieved with newer protocols. In our hands, activation of 25 million T cells is sufficient to consistently achieve therapeutic CAR-T cell doses within a 7-day protocol. This opens a new realm of possibilities, as manufacturing could potentially be possible from a single regular blood draw.^9^

##### Bulk PBMCs

Using bulk PBMCs from leukapheresis as starting material for CAR-T cell production is cost effective and simple, but platelet and red blood cell (RBC) contamination can compromise in-process testing and are often associated with aggregation, poor T cell expansion, and low viability.^10^^,^^11^^,^^12^ Several processing devices can perform density-based cell separation, cell washing and concentration, and platelets or RBC removal (Table 1).^13^ A detailed review of the currently available wash-and-concentrate devices is beyond the scope of this review.^14^

**Table 1:** Cell processing devices that can be used in preparation of starting material for CAR-T cell production

Device	Capabilities/protocols
COBE 2991	• red blood cell (RBC) washing
	• deglycerolized frozen RBCs
	• preparation of leukocyte concentrates
	• collection of young RBCs
	• bone marrow processing using a density gradient separation medium
	• bone marrow concentration
	• mononuclear cell washing
	• cell concentration
	• large volume wash
Sepax C-Pro	• PeriCell (concentrate cells from fresh apheresis product via plasma depletion)
	• platelet free (concentrate cells and remove platelets from fresh apheresis units)
	• BeadWash (incubate magnetic beads onto cell fractions obtained from apheresis units—sequence concentration, platelet depletion, incubation, and washing)
	• NeatCell (enrich mononuclear cell fraction via a density gradient medium)
	• SpinOculation (concentrate, wash, and transduce isolated cells by spinoculation)
Rotea	• low-volume recovery
	• small- to mid-scale cell processing
	• T cell, NK cell, and MSC wash and concentration
	• cell wash/buffer exchange
	• iPSC aggregate processing
	• PBMC/monocyte separation
	• elutriation and cell separation
	• RBC depletion
	• residual wash out
	• QC sample prep and isolation
	• MSC harvesting from cell factory systems
	• lentiviral vector clarification
LOVO	• immunomagnetic selection
	• fresh leukapheresis wash
	• culture harvest and media exchange
	• thawed wash and DMSO removal

Information extracted from manufacturer’s websites and published data.^14^^,^^131^^,^^132^^,^^133^

##### Purified T cells

Monocyte and macrophage content in the leukapheresis starting material can compromise CAR-T cell manufacture through inhibition of T cell expansion^15^ and, in patients with high levels of circulating disease, CAR transduction of B cell acute lymphoblastic leukemia (B-ALL) blasts has been associated with leukemia relapse due to epitope masking.^16^ These risks can be mitigated through a T cell enrichment step, frequently via immunomagnetic cell separation. In our experience, despite an increase in costs, this has resulted in important improvements in product consistency and has reduced manufacturing failure rates.

Lonza has recently incorporated a magnetic selection module into the automated, closed-system Cocoon platform. This allows the selective enrichment and activation of T cells using Dynabeads CD3/CD28.^17^

Miltenyi’s CliniMACS Cell Separation technology was introduced three decades ago^18^ and the CliniMACS Plus device is now a well-established system for automated and current GMP (cGMP) immunomagnetic cell enrichment or depletion for hematological progenitor cells grafts^19^^,^^20^ and other applications.^21^^,^^22^^,^^23^ This cell selection technology is incorporated into the CliniMACS Prodigy device, an all-in-one solution comprising fully automated cell enrichment and CAR-T cell manufacture including transduction and T cell expansion.

##### T cell subsets with preferred phenotypes

Selective enrichment of preferred phenotypes has many potential advantages for the development of CAR-T cell products with optimized efficacy and potency. For example, CD19 targeting CAR-T products with defined CD4:CD8 ratios have been shown to display superior antitumor reactivity and *in vivo* efficacy,^24^^,^^25^ but the manufacture of two separate products for mixing at a given ratio significantly increases costs.

CAR-T manufacturing from purified central memory T cell (T_CM_) or stem-like memory T cell (T_SCM_) populations also has the potential to enhance antitumor responses.^26^ Pre-enrichment of CD62L+ cells has been included in some protocols and can be easily achieved with the CliniMACS system.^27^ However, selection of complex phenotypes would require multiple selection stages, which can become prohibitively expensive and impractical. Turtle et al.^25^ achieved efficient selection of CD8+ TCM cells using a sequential two-step enrichment including a CD4+/CD14+/CD45RA+ depletion step, followed by CD62L+ positive selection.

Fluorescence-activated cell sorting (FACS) allows multiparameter sorting with much higher purity and can be used when requirements are too complex for adequate results to be achieved with available magnetic-based separation technologies.^28^ However, most of the currently available sorting systems are not fit for large-scale clinical product manufacture as they are low-throughput devices, with non-sterile fluidics systems and not GMP compliant. Closed-system devices are being developed by multiple companies and should fill in this market gap in the upcoming years. The MACSQuant Tyto allows ten-parameter cell sorting with the use of closed-system cartridges. Unlike with droplet sorters, cells are not subjected to high pressure, decompression, and charges, maintaining sample viability. This technology has been increasingly employed, especially in the field of CAR Tregs.^29^^,^^30^ Other recently released FACS-based cGMP sorting technologies include the Sony GMP-ready CGX10 Cell Isolation System. This device uses a proprietary microfluidic chip and a built-in temperature control cabinet, enabling fully closed multiparametric, fluorescence-based cell sorting.^31^

#### Activation

Polyclonal T cell activation is generally required for effective transduction with lentiviral and retroviral vectors and is frequently achieved using CD3 and/or CD28 agonist antibodies. In soluble form these antibodies can only support short-lived T cell activation, but when immobilized they can induce signaling similar to that observed in antigen presentation, resulting in sustained TCR-mediated activation and productive T cell responses.^32^ Immobilized CD3/CD28 antibodies are available in GMP grade in multiple forms.

CTS Dynabeads (CD3/CD28 magnetic beads) provide T cell isolation and activation within a single reagent. However, one limitation of this approach is the requirement for removal of the magnetic beads at the end of the manufacturing process, which is labor-intensive if done manually, and can lead to considerable cell losses.^33^ The integrated magnetic separation feature of the Cocoon platform allows automated bead removal, but this is not compatible with the Miltenyi MACS technology, which limits availability of GMP-grade magnetic labeling reagents.

On the other hand, the MACS GMP T cell TransAct activation reagent is a biodegradable polymeric nanomatrix, and does not require removal prior to end of process product formulation. The supplier confirms that the reagent offers no safety concerns by day 7 of a typical manufacture process.

Expamers are polymeric, soluble protein complexes, formed by anti-CD3/CD28 Fab fragments linked to a recombinant Streptactin backbone and designed to activate human primary T cells without the use of solid support while still providing the required contact surface areas. In principle, this soluble reagent could be compatible with any manufacturing platform, and termination of the activation signal can be instantly triggered with addition of non-toxic D-biotin.^34^ The ability to fine-tune activating signals may have an important role in the determination of T cell fate and may be an important tool for manufacture of products with more favorable phenotypes,^35^^,^^36^^,^^37^ although this remains to be evaluated in a clinical setting.

The ImmunoCult Human CD3/CD28 T cell Activator (STEMCELL Technologies) consists of soluble CD3 and CD28 antibody complexes and is available as a GMP compliant reagent for use as an ancillary material in clinical manufacture. This reagent has been demonstrated to be efficient in promoting T cell expansion in the presence of IL-2.^38^^,^^39^ Research efforts are currently focused on novel alternative activation approaches (including engineered viral vectors) and non-activation protocols. LentiSTIM combines activation and transduction steps using viral particles with envelope anti-CD3 and anti-CD28 membrane-bound mitogens, incorporated into their viral envelope and derived from the membrane of producer cell lines.^40^ The single activation/transduction step may be beneficial to reduce costs and manufacturing times, but its use in clinical-grade manufacturing remains to be evaluated. Alternative activation methods, such as co-stimulation via the CD27 axis has been shown to maintain memory phenotype and improve therapeutic activity of CAR-T cells^41^ and, more recently, optimized protocols designed to preserve stemness of T cells have explored removal of the activation step^42^ altogether. These approaches have not yet been tested in GMP manufacturing practice.

##### Expansion

A diverse range of platforms are available for expansion of suspension cells, varying in terms of capacity, automation level, flexibility of protocols, costs, and integration with external devices for monitoring and/or further processing (Figure 1).

Cell culture bags are available from a range of suppliers as a closed-system alternative to traditional culture flasks. They are inexpensive consumables, but protocols are fully manual, requiring multiple feeds and media exchanges depending on cultivation length. Taking into account the requirements for staff and cleanroom space, two major bottlenecks in current cell and gene therapy manufacturing, cell culture in bags can be impractical in many centers. For this reason, many of the initial CAR-T manufacturing protocols included an initial cultivation in bags, transduction in retronectin-coated bags, but then transfer into a bioreactor for expansion.^7^^,^^43^ Although the multiple bioreactors and automated devices available in the market can have different footprints and requirements in terms of cleanroom space and operation, they can typically be used in lower-grade environments due to closed system operation (e.g., grade D environment in EU GMP as opposed to a grade A environment with a grade B background, as required for open handling steps), they allow parallelization of the production process, and automation of washes and feeds releases staff for execution of other critical processes, therefore allowing an increased manufacturing capacity within a limited cleanroom space and reduced workforce, as is the case in many academic centers.

As an alternative to culture bags and traditional flasks, the G-Rex flasks have a unique gas-permeable membrane and an optimized volume/area ratio, which allows cells to grow completely undisturbed for several days, with no need for feeds, media exchanges, or mechanical motion. The flasks are available with a wide variety of working volumes (100 mL, 500 mL, 1 L, 5 L) and a recommended seeding concentration of 500,000 cells per cm^2^. They can be used in association with a peristaltic pump, which allows volume reduction and cell harvest within a closed system. However, although the use of the G-Rex culture system can decrease the requirement for manual handling, they still require the use of CO_2_ incubators and they can only be used for the expansion stage, with seeding, washing, and harvest having to be performed manually, and no integration for the remaining manufacturing steps. Even so, due to its flexibility, adaptability, and cost-effectiveness, the G-Rex system is widely employed for a variety of cell therapy applications.

WAVE bioreactors, and now the Cytiva’s Xuri Cell Expansion System, are cell bag bioreactors, with capacity of up to 25 L. Single-use bags are kept in a heated rocking base, designed to inflate and shake for optimal gas dispersion, and media can be added within a functionally closed system in continuous perfusion or fed-batch mode. Process parameters such as rocking speed, dissolved oxygen, pH, and perfusion rate can be continuously monitored and controlled. These systems are well-established as effective platforms for expansion of CAR-T products and can accommodate a broad range of cell numbers.^44^ However, the lack of integration with other devices for enrichment, cell wash, and transduction hinder their application.

The Lonza Cocoon is a stand-alone cell-processing device, with a range of capabilities including cell selection, activation, transduction/transfection, expansion, and harvest in an automated and functionally closed system. Protocols can be flexibly developed to be fully integrated using customizable cassettes, tailored to the specific needs of each product. The integrated magnet for cell enrichment is compatible with the use of Dynabeads, but labeling needs to be performed manually or with the use of other devices. Due to media recirculation and continuous monitoring of CO_2_, dissolved oxygen, and pH levels, cell expansion can be performed with no need for counting and re-seeding, but culture volumes and cell yield are limited. Optimal seeding numbers are reported between 50 and 100 × 10^6^ cells. The Cocoon platform also has the advantage of integration with the Lonza 4D-Nucleofector, a device that has been extensively validated for manufacture of cell and gene therapy products,^45^^,^^46^^,^^47^ therefore allowing the easy implementation of non-viral gene delivery protocols.

The CliniMACS Prodigy is another all-in-one alternative that provides automation of all steps of the CAR-T manufacturing process. This device uses the well-established MACS technology for cell enrichment, allowing fully automated cell washing, labeling, and magnetic separation within a closed system. The system comes with pre-programed and validated application-specific processes, including a T cell transduction protocol that allows flexibility in terms of starting material enrichment, definition of transduction and cultivation timelines and volumes, as well as pattern of mechanical agitation. Cells are grown within the device’s cultivation unit from a starting number of up to 200 × 10^6^ in the standard version, in a maximum volume of 300 mL and at a set temperature and CO_2_%. Furthermore, the device can be readily employed for applications requiring the use of genome-editing tools and non-viral transgene delivery thanks to the availability of an electroporation module that can be integrated for closed-system processing.

However, sensing and monitoring capabilities are limited, and media exchanges must be programmed by the operator according to cell expansion profile.

##### Formulation and cryopreservation

Automation for final formulation and cryopreservation is one of the main unmet technical needs in the cell and gene therapy field. Although aseptic filling devices are common in the pharmaceutical industry, cell product requirements are highly variable in terms of final composition, volumes, cell numbers, and types of containers, therefore a one-size-fits-all solution may be difficult to achieve. Furthermore, cellular products are especially delicate and require careful handling. Critically, extended exposure to cryoprotectants containing DMSO is toxic to the cells, therefore products must be kept cold and quickly cryopreserved after final dilution.^48^ For these reasons, the final formulation and cryopreservation of CAR-T product is still performed manually in most centers.

Terumo’s FINIA Fill and Finish System is a closed, automated system that formulates and aliquots cell suspensions to prepare for cryopreservation. Cells and cryoprotectant solution are mixed in a cooled bag and automatically dispensed into up to three dosages and one QC sample in cryobags with volumes set by the user. Cryobags are sealed and ready for cryopreservation in approximately 10 minutes, but the system cannot dispense low-volume doses or work with vials, which remains an unmet need.

Another fluid-handling platform for closed system bioprocessing is Sexton’s Signata CT-5TM. This device uses a peristaltic pump for mixing, rinsing, and filling of cryobags or closed-system cryogenic vials, such as CellSeal vials. The Signata CT-5TM has a smaller footprint than the FINIA, but the process is not fully automated.

Recently launched, the ScaleReady’s Cue system uses syringe pumps, a valving cassette, and a spinning membrane separation technology, to enable high accuracy concentration, formulation, and aliquoting in a functionally closed system. One advantage of this system is the accurate handling of low volumes, supporting final product volumes as low as 2 mL.

Finally, Miltenyi recently released a formulation unit for use with the CliniMACS Prodigy. This attachment allows the cells harvested at the end of a typical T cell transduction process to be resuspended at the correct concentration in the final formulation medium prior to addition of cryoprotectant and subsequent dispensing of defined volumes into cryobags. However, the flexibility of the application is limited, and the device is only suitable for filling of higher volumes in bags, while low volumes and vials would still need to be manually filled.

##### Future of automation and considerations for phase 1 trials

Given the rapidly growing number of CAR-T products advancing into commercialization and the limited availability of cleanroom space and trained personnel, automation solutions are still required for higher throughput and scaling out to meet the demand of over 100,000 CAR-T batches over the next 10 years for the European market alone.^49^ Inspiration can be found in other well-established industries, such as the automotive, food, and classical pharmaceutical industries, for which fully automated manufacturing plants are the norm.^50^

Beyond manufacturing systems, consideration must also be given to the quality control aspects. Batch release testing minimally evaluates quantity (live cell counts), identity (CAR expression), purity (e.g., presence of endotoxin or residual activation beads), and safety (microbiological assessment). Samples are manually processed, and results may take up to 2 weeks, such as in the case of culture-based methods for sterility assessment.

To upscale manufacturing capacity, two main strategies can be employed: (1) the use of parallel all-in-one systems, working simultaneously or (2) modular integration of diverse platforms, each performing specific sub-processes of the CAR-T manufacturing protocol. While the first approach may be more easily available based on current technology, the latter certainly optimizes the use of space and resources and is the most commonly adopted approach in modern assembly lines in different industries. However, full automation will require better integration between different devices, as well as implementation of new real-time data monitoring and processing systems. Fully automated, smart manufacturing plants, using real-time data analysis, with digital communication of all devices and integration of concepts of machine learning to further minimize the need for human intervention are a reality within the Pharma 4.0 concept and have the potential to make CAR-T cell therapies more accessible. All these technical innovations must be adaptable to account for the biological advancements in CAR immunotherapy and they must be in line with the requirements of regulatory agencies.

Setting up fully automated smart plants is only feasible if processes are demonstrated capable and well defined, and proven to be suitable under a broad range of conditions. This is possible with an extensive characterization of products and processes in the development phase, with the assistance of a quality by design (QbB) approach.^51^ In the case of early phase development and manufacturing for phase 1 trials, it is common that critical quality attributes are still being defined. Therefore, processes must be flexible enough to be fine-tuned as data become available and models are improved. Furthermore, early-stage clinical trials typically recruit a small number of patients. Therefore, for phase 1 trials the focus is usually evaluating a number of different products, but a small number of batches for each, which makes flexibility a fundamental requirement of any platforms used. In the academic setting, process development for phase 1 trials should focus on evaluation of technologies and on the optimization of manufacturing processes to reliably generate the best CAR T products. Given that these are mostly first-in-human studies, where cutting edge developments are evaluated, the use of research-grade reagents and platforms are generally deemed acceptable by regulatory authorities, where no GMP alternatives exist (e.g., EU GMP guidelines in EudraLex, volume 4, part IV, 7.13).

However, because changes in the manufacturing processes can be cumbersome when moving to commercialization, processes must be developed taking into account scaling-out for later stages. This may require early discussions and collaboration with suppliers, and automation has the advantage of facilitating technology transfer between different manufacturing sites.

### Improvement of product phenotype

It is clear that CAR-T persistence is critical to ensure sustained remission. Determinants of response and resistance to CAR-T cell therapy have been sought by multiple groups and, although highly dependent on engineered construct, such as the choice of co-stimulatory domains,^52^ sustained responses for the same product have been demonstrated to be associated with specific phenotypes, such as the frequency of early memory subsets, with an oxidative phosphorylation (OXPHOS) directed metabolism and low expression of exhaustion and senescence markers.^53^^,^^54^ Importantly, CAR-T cell manufacturing strategies can have a significant impact on final product phenotype and should be evaluated with care.

The differential capacity of CD4 and CD8 CAR-T cells to proliferate and persist has been demonstrated *in vivo*^24^ and their respective roles in initial and long-term responses may differ*.* These two subsets appear to exert synergistic activities with respect to expansion and antitumor activity^24^ and the benefits of CAR-T products with a fixed CD4:CD8 ratio have been evaluated.^55^ In this regard, manufacturing protocols may have a critical role in determining efficacy by influencing predominance of each subset in the final drug product. Turtle et al. have demonstrated that the frequency of CD4 and CD8 T cells is substantially different between patients and healthy donors, with often an inverted CD4:CD8 ratio in heavily pre-treated patients. Beyond selective enrichment in the starting material, the frequency of each subset can also be influenced by activation method and culture conditions. For example, anti-CD3/CD28 beads, the activation method of choice for most manufacturers, are known to favor CD4 expansion over CD8.^24^^,^^56^^,^^57^ On the other hand, the use of soluble CD3-targeting activation methods can have the opposite effect.^56^ This is likely associated with the differential activation threshold of each subset, as duration of stimulation for optimal T cell expansion has been demonstrated to differ between CD4 and CD8 T cells.^58^ While CD4 expansion is favored upon sustained antigen exposure, CD8 T cells are able to proliferate and differentiate into effector T cells in response to transient antigen presentation, but rapidly become exhausted, or activation-induced nonresponsive.^59^

As manufacturing data accumulate, the development of models to predict final phenotype based on starting material characteristics and culture conditions have emerged as potential solutions to some of the manufacturing hurdles associated with the variability of autologous patient material.^60^ This can be an alternative to the expensive approaches used today to obtain a fixed ratio of CAR-T cell products by optimization of manufacturing protocols in a patient-specific manner to determine the activation method and culture conditions best suited for the starting CD4:CD8 ratio, considering variables such as differentiation stage and response to antigen exposure and cytokines.

In our experience, T cell activation using TransAct CD3/CD28 beads for 4 days, followed by cultivation in a combination of IL-7 and IL-15 can favor expansion of CD4+ cells, with final products often having a skewed CD4:CD8 ratio.^61^ However, even if under-represented in the infused product, CD8+ cells tend to expand quickly *in vivo* in response to antigen stimulation and typically represent the majority of CAR+ cells detected in the patient’s blood at early follow-up,^62^ with a lower CD4/CD8 ratio in the blood at days 6–41 post infusion being associated with improved responses.^63^ This dynamic feature complicates the assessment of the individual contribution of each T cell subset in the final products to clinical responses.

It is also increasingly clear that therapy efficacy is linked to the differentiation stage of the cells infused, with naive (T_N_), T_CM_, and T_SCM_ lymphocytes related to a better long-term response due to their ability to proliferate and persist longer.^64^ Therefore, strategies to develop products enriched in these compartments and to uncouple T cell expansion from differentiation are constantly being sought. Beyond introduction of shorter or no-expansion protocols, which are discussed in more details below, T_N_ and/or T_SCM_ can be enriched for CAR-T cell manufacture via multi-stage magnetic selection^24^ or FACS.^26^^,^^65^

Activation method, strength, and duration of the stimulus seem to be critical in determining final product phenotype. Anti-CD3/CD28 bead-treated T cells typically assume a T_EM_ phenotype by day 14, but transient stimulation with antigen-presenting cells has been shown to provide superior expansion of CD8 T_SCM_, while improving cytokine secretion, anti-tumor effects, and *in vivo* persistence.^58^ Furthermore, earlier wash and removal of activation beads has been reported beneficial for T cell expansion in automated manufacturing protocols.^66^

Composition of the cultivation medium, presence of serum or sera substitutes, and exogenous cytokines have a determinant role in final product phenotype but are complex to evaluate. Small adjustments to media formulations, such as an increased potassium content, have been described to preserve T cells in a stem-like state where they retain the capacity to expand.^67^ On the other hand, while IL-2 supplementation has been common practice to support cell expansion, it can drive differentiation into T_EM_ and favor proliferation of T_regs_, which can hinder therapy efficacy.^68^ New combinations of cytokines including IL-7, IL-15, IL-18, and IL-21 are becoming increasingly common.^1^

Overall, improving product phenotype for sustained responses will depend on a profound understanding of T cell biology, signaling, and immunological memory. Understanding the mechanisms associated with the transformation of quiescent naive cells into fast-proliferating effectors, and then again into quiescent memory cells, provides key information on what are the determining features of a good product and, therefore, which cellular processes can be rewired for better phenotype. We know that T cell activation is coupled to a shift in cell metabolism from basal OXPHOS into a highly metabolically active state using glycolysis to support the energy requirements for rapid expansion.^69^ Conversely T cell stimulation in the presence of glycolysis inhibitors can enhance the generation of memory cells and improve antitumor activity.^70^ On the other hand, differentiation into memory T cells requires entry into a primed state, where cells remain quiescent, but can rapidly respond to a previously encountered antigen. This is associated with a shift toward fatty acid oxidation and OXPHOS and a higher mitochondrial reserve and increased levels of cardiolipin.^69^ The impact of different metabolites of cultivation medium and tumor microenvironment on epigenetic signatures and determination of T cell fate has been reviewed by Akbari et al.^71^

Induction of a less-differentiated phenotype can also be induced with the use of pharmacological inhibitors of critical T cell signaling pathways such as PI_3_K/AKT/mTOR to uncouple T cell proliferation from differentiation and exhaustion.^72^^,^^73^^,^^74^^,^^75^ Similar results have been demonstrated with the use of ibrutinib to block interleukin-2-inducible T cell kinase/Bruton’s tyrosine kinase signaling during autologous manufacture for chronic lymphocytic leukemia patients.^76^ Similarly, addition of dasatinib during manufacture, a clinically available tyrosine kinase inhibitor that inhibits essential proximal CAR signaling kinases, has been shown to prevent exhaustion of CAR-T cells and has been implemented in phase 1 trials.^77^^,^^78^ Despite the advantages of these tools, it is important to mention that inclusion of additional components in the cultivation medium can be a logistical and regulatory burden, especially if the product is taken beyond phase 1. The regulatory status of these drugs, which need to be manufactured under GMP compliance and qualified as suitable for their use as ancillary materials, must be taken into account. A close interaction with suppliers from the earliest stages of development is essential to ensure that the correct grade material will be available for large-scale production. Furthermore, residual levels should be carefully evaluated to minimize risk to patients.

*In vivo*, repeated exposure to antigen and tonic signaling are associated with terminal differentiation and exhaustion. This has been demonstrated to be determined by CAR design, especially the choice of co-stimulatory domains.^78^^,^^79^ For example, whereas CD28-co-stimulated CARs are often described to have better effector function, persistence, and long-term responses are poor when compared with 4-1BB-co-stimulated CARs, which tend to exhibit a memory-like phenotype.^79^^,^^80^ This is associated with strong activation mediated by CD28ζ, which drives rapid T cell differentiation and high effector function preceding T cell contraction.^81^ Other contributing factors include CAR expression levels, promoter strength, hinge, and transmembrane domains and scFv characteristics. For example, we have demonstrated that a lower-affinity CD19 binder leads to enhanced expansion and persistence of CAR-T cells post-infusion,^43^^,^^61^ which is associated with maintenance of a T_SCM_ phenotype.^82^^,^^83^ Changes in manufacturing protocols can also be implemented to prevent loss of function due to excessive tonic signaling, which is often associated with clustering of receptors in the plasma membrane.^80^ Optimal CAR expression levels can be achieved with choice of the correct expression platform^84^ and fine-tuning of multiplicity of infection used for viral vectors. Clustering of receptors can also be prevented by regulating the ionic strength of the cultivation medium, with a high-salt treatment significantly reducing the tonic signaling index.^85^

Furthermore, regardless of product phenotype at infusion, the harsh immunosuppressive and anaerobic tumor microenvironment can lead to T cell exhaustion and senescence,^86^ a concern particularly in the case of solid tumors. Engineering strategies are being evaluated now by many teams and can include PD-1 knockout using Crispr-Cas9,^87^ the use of a dominant negative TGF-β receptor to block TGF-β signaling^88^ co-expression or secretion of cytokines, such as IL-7 or IL-15,^89^^,^^90^^,^^91^ use of constitutively active receptors^92^^,^^93^ or hypoxia-activated CARs with the use of an HIF-1a subdomain.^94^ Although some of these approaches rely on the use of multicistronic vectors and therefore change little of the manufacturing requirements, quality control becomes increasingly complex and potency characteristics challenging to evaluate. With the use of genome-editing tools, features like efficacy of gene editing, genotoxicity, tumorigenicity, and immunogenicity are critical quality attributes that need to be evaluated during development and/or at batch release.^95^

### Rapid manufacturing protocols

The applicability of CAR-T cell therapy is still limited by manufacturing capacity worldwide, and strategies to decease vein-to-vein time are highly desirable. Beyond decreasing patient wait times, rapid manufacturing protocols also have the potential advantage of limiting the impacts of *ex vivo* expansion on product quality.

Early cell therapy manufacturing protocols, such as those developed for tumor-infiltrating lymphocyte therapy (TILs) in the 1980s, required extended *in vitro* expansion with high doses of IL-2, aiming to achieve therapeutic doses of hundreds of billions of cells.^96^ While effective doses for CAR-T cell therapies have been established to be 1,000-fold lower than for TILs, at least for hematological malignancies,^97^ early CAR-T manufacturing protocols still involved between 9 and 14 days of expansion,^7^ leading to progressive T cell differentiation in culture. To investigate whether this period could be shortened, Ghassemi et al.^98^ demonstrated that products harvested from culture between days 3 and 5 exhibited less differentiation and enhanced effector function *in vitro* when compared with the standard 9-day protocol, highlighting the potential importance of curtailing the *in vitro* manufacturing period.

The feasibility of a next-day manufacturing protocol, where T cells are enriched and activated with CD3/CD28 Dynabeads, transduced after 24 h with a CD19 CAR-encoding lentiviral vector, and frozen the next day with no expansion step, has been evaluated in a phase 1 trial (NCT03825718). These products exhibited better *ex vivo* expansion upon stimulation with CD19-expressing cells and a less-differentiated and less-exhausted phenotype.^99^ Furthermore, when treated with low doses, ranging from 10^4^ to 10^5^ cells/kg, 23 out of 25 adult and pediatric B-ALL patients achieved minimal residual disease-negative remission on day 28 after infusion. However, because most patients underwent allogeneic hematopoietic stem cell transplantation within 3 months post CAR-T cell therapy, evaluation of long-term responses was not possible.

On the commercial side, Novartis announced T-Charge as their next-generation CAR-T manufacturing platform. Here, T cells are enriched, activated, and transduced on the same day with a lentiviral vector encoding the same CD19-targeting CAR construct used for tisagenlecleucel, and then harvested, washed, and formulated after 2 days in culture. When tested clinically at doses 25 times lower than tisagenlecleucel (which is manufactured in a typical 10-day process), this platform provided products with maintained T cell stemness and with promising overall safety and excellent CAR-T cell expansion.^100^

An alternative approach for rapid CAR-T manufacture described recently involves the optimization of lentiviral transduction of non-activated T cells, aiming to further reduce the irreversible differentiation of T cells triggered by T cell receptor activation with CD3/CD28 antibodies.^42^ Cells manufactured with this protocol were demonstrated to be potent *in vitro* and *in vivo*, but transduction efficiency is still suboptimal when compared with the standard activated products.

The use of no-expansion protocols is currently limited by costs of viral vector required for transduction of a large cell number. However, since data so far indicate that non-expanded cells may have a superior phenotype, it is likely that effective doses can potentially be significantly lower.

Lastly, prior to widespread adoption of rapid manufacturing methods, the effective removal of residual activation beads and free viral particles, as well as other impurities in the final formulated product, needs to be carefully evaluated. Extensive washes are likely to be required and a rigorous evaluation of the risks associated with residual bound beads is needed to ensure product quality and patient safety.

### Genome-editing technologies

Transcription activator-like effector nucleases and clustered regularly interspaced short palindromic repeats-Cas9 (CRISPR-Cas9) now offer powerful genome-editing tools to create allogenic, off-the-shelf CAR-T products,^101^^,^^102^ with locus-specific insertion of the CAR transgene^103^ and the potential for multiplexed editing to manipulate critical T cell signaling pathways.^104^ The role of CRISPR-Cas9 in the development of next-generation CAR-T therapies has been reviewed in detail by Dimitri et al.^105^

Targeted delivery of GMP-grade editing components (in the form of proteins, DNA, and/or RNA) to T cells is a critical step and is most frequently achieved using electroporation. This is efficient and versatile, but can lead to significant cell death and may not be suitable for all cell types.^106^ Pulse format, duration and intensity, as well as cargo concentration and ratios need to be carefully optimized at the pre-clinical stage. Closed-system, GMP-compliant electroporation devices are now available in the market. The Lonza 4D-Nucleofector has a large-scale unit with a single-use weldable tubing-set that can be connected to the Cocoon system. Miltenyi has also released the CliniMACS Electroporator, which can be connected to the CliniMACS Prodigy for automated transfection. Lastly, MaxCyte has also released the ExPERT GMP Processing Assemblies, which allow large-scale flow electroporation in a closed system.

Lipid nanoparticles (LNPs) have been demonstrated to be an effective, and likely gentler, delivery method for *ex vivo* gene editing,^107^ with *in vivo* clinical efficacy demonstrated over the past 2 years with COVID-19 mRNA vaccines.^108^ These LNPs can be generated and loaded with mRNA cargo using a variety of chemical methods,^108^ but microfluidic mixing has the advantage of allowing precise control of particle size, high reproducibility, and high encapsulation efficiency.^109^ Furthermore, this method is scalable and can be automated for clinical production. The NanoAssemblr platforms (Precision Nanosystems) can formulate mRNA-loaded LNPs with a variety of sizes and compositions, with a choice of devices suitable for formulation volumes from 25 μL to >10 L.

However, while genome-editing platforms offer solutions augmenting CAR-T cell therapies, they are not without drawbacks. Multiple disputes on intellectual property ownership are centered around the CRISPR-Cas9 technology, and the use for human applications may require complex licensing agreements.^110^ Furthermore, from a manufacturing perspective, gene editing is a complex procedure that requires highly skilled operators and adds significant costs to CAR-T cell products that are already expensive, which may prove a bottleneck for any products to progress beyond phase 1.

Gene editing is associated with genotoxicity, linked to double-stranded breaks, insertion and deletions (or INDELS),^111^ bystander and off-target edits,^112^ and chromosomal aberrations.^113^^,^^114^^,^^115^ For this reason, advanced, and sometimes costly, quality testing procedures need to be implemented to ensure the safety of gene edited CAR-T cell products. During product development, the genome-editing components and protocols must be optimized and verified not only to ensure functionality of the edited cells and the intended downstream biological modifications but also to minimize off-target effects.^116^^,^^117^ Assessments of off-target editing frequency, interchromosomal and intrachromosomal rearrangements, and residual editing components are also required. This typically includes a combination of genome-wide analyses (e.g., *in silico* or cellular-based assays) and verification of sites identified using methods with adequate sensitivity to detect low-frequency events.^118^ Release testing also includes evaluation of on-target editing efficiency.

Recently, base-editing technology has emerged as a potentially safer alternative to traditional CRISPR-Cas9. In brief, instead of the double-strand breaks promoted by Cas9, this system uses a catalytically disabled nuclease fused to a deaminase enzyme that is capable of mediating highly precise base conversions, which can be directed to precise positions to create premature stop codons or disrupt splice sites.^119^

Lastly, although GMP manufacture of RNA can be prohibitively expensive, significant progress has been made over the last few years with the use of circular RNA (circRNA). Compared with linear mRNA, circRNA would appear to be easier to manufacture and may potentially be used in much lower concentrations. In one exemplar preclinical CAR-T manufacturing study, base editing was used for increased PD-1 knockout efficiency.^47^

## Quality control and release testing

As CAR-T cell products increase in complexity, development of adequate quality control assays becomes a critical challenge. It is essential that enough pre-clinical data are available to allow determination of the main critical quality attributes of each product and the use of testing methods that are fit for purpose from the offset, so that appropriate product specification can be defined. The regulatory requirements for release testing depend on the phase of clinical development. For phase 1 trials, regulatory bodies accept that assays may still require optimization; however, minimum requirements in terms of safety, identity, purity, and potency must be met. Typical quality control requirements for CAR-T cell products are described in Table 2.

**Table 2:** Examples of CAR-T cell quality control requirements

Parameter	Tests
Quantity	• total T cell count and CAR-T cell numbers
Viability	• T cell viability
Safety	• sterility and mycoplasma detection
	• RCL/RCR
	• vector copy number
	• for gene-edited products: off-target effects, chromosomal abnormalities
Identity	• CAR surface expression
Purity	• cellular composition and transduction efficiency
	• assessment of process-related impurities (e.g., residual activation beads)
	• endotoxin levels
Potency	• target-directed cytotoxic activity
	• target-induced IFN-γ secretion

Often, product testing represents the biggest bottleneck for delivery of autologous therapies. Culture-based sterility assessment methods typically take 10–15 days to produce results, limiting the turnaround times even if rapid manufacturing methods are employed. More importantly, a limited number of licensed testing sites are available in Europe to carry out more complex assays, such as detection of replication-competent lentiviruses and retroviruses, or high sensitivity detection of off-target effects following the use of genome-editing tools, and backlogs can cause significant delays to product delivery. Therefore, it is essential that manufacturing advancements are accompanied by the implementation of automated and streamlined testing methods, developed in accordance with the International Council for Harmonization (ICH) of Technical Requirements for Pharmaceuticals for Human Use guidelines and demonstrated to be robust and appropriate for the specific stage of development. The latest innovations applicable to the testing of CAR-T cell products are discussed below. Many of these new technologies are being developed by the biotechnology industry, and their evaluation in the academic setting in the context of early-stage clinical trial offers many advantages. While this model of collaboration allows generation of datasets that can be used for validation and demonstration of suitability for the intended application, it also allows an extended characterization and a better understanding of the products through the use of innovative methods.

### Safety

Safety assessment minimally comprises assays to ensure that the final product is free from microbial contamination, mycoplasma, and impurities, such as endotoxin.

Sterility assessment remains a major obstacle to rapid release of CAR-T cell products. The introduction of colorimetric and fluorescence-based CO_2_ measurements of metabolic activity (e.g., BacT/Alert 3D and BD BACTEC systems) or adenosine triphosphate detection by bioluminescence (Rapid Milliflex Detection System) has allowed for faster evaluation of microbiological contamination. However, a minimum of 7 days incubation at 35°C–37°C is still recommended for an accurate assessment.

Rapid sterility testing methods have recently become available, such as the Microsart ATMP Sterile Release kit (Sartorius). This is a PCR-based assay that uses TaqMan probes for detection of a broad range of Gram-positive and Gram-negative bacterial and fungal contaminants (results are obtained within 3 h; therefore implementation of this assay would allow for rapid release of ATMP products). DNA-based methods are already widely employed for detection of mycoplasma specimen in CAR-T cell products. However, because these methods require extended sample handling for DNA extraction and preparation of the qPCR assay, risks of cross-contamination must be considered carefully and mitigated accordingly. Sample handling in a sterile, DNA-free environment is essential, with the use of isolators recommended by the manufacturer.

Safety evaluation of cell therapy products has gained a whole new dimension with the introduction of genome-editing tools. Off-target effects need to be carefully evaluated at the development stage to ensure guides designed are optimal. This can be done with the use of prediction tools, but also experimentally with off-target profiling methods for whole-genome analysis, including GUIDE-seq, DIGENOME-seq, CIRCLE-seq, or SITE-seq. The variety of methodologies and their advantages have been reviewed previously.^120^ Assessing breaks or deletions at these potential off-target sites, as well as the desired editing event, requires the use of whole-genome sequencing of the edited products and amplification of the candidate off-target sites, followed by deep sequencing to provide adequate sensitivity for low frequency events.^118^ In case of methods that induce double-strand breaks, such as CRISPR-Cas9, the risk of large deletions or insertions, inversions of gene fragments, and chromosomal translocations is also a concern and should be evaluated.^121^

### Identity and quantity

The main purpose of identity assessment is to define the cellular composition of the final products, particularly the expression of the CAR. Detection of the CAR can usually be performed by flow cytometry, and it can either detect the CAR itself or surrogate marker genes. Direct detection of the CAR molecule is performed using CAR-specific reagents: these can either be an antibody specifically raised against the binder, antibodies against the stalk region (such the IgG portion), or the antigen-Fc detection proteins. Absolute quantification can be obtained with the use of counting beads or with the use of a dual platform approach, where absolute cell numbers are obtained with the use of automated hematological counters. As flow-based detection assays are mostly CAR specific, the method needs to be optimized and validated for each cell product, ideally demonstrating specificity, precision, accuracy, linearity, sensitivity, and range.^122^ Traditional flow cytometry methods can be variable, and results are often operator dependent, which is often an issue in the GMP setting. It is critical that the use of an optimized method is allied with standardized instructions for gating and adequate training. Although the custom nature of these detection assays represents a challenge for the setup of multicenter proficiency programs, some of these concerns can likely be addressed with the help of automated analysis tools, currently under development, which use a variety of unsupervised and supervised machine learning algorithms to automatically identify populations of interest.^123^ Furthermore, automated sample preparation workstations are now available for integration with a variety of devices, such as the BD FACSDuet for the BD FACS Lyric, the Biomek i5 for the Beckman Coulter Cytoflex, and the Sysmex PS-10. Lastly, cartridge-based technologies are also in implementation for product phenotyping, as is the case of the Accellix platform.

#### Purity

Process-related impurities include residual components of the cultivation media and buffers, selection or activation beads, viral vectors, enzymes, and DNA and RNA used for genome editing. Typically, these impurities are removed with the inclusion of extensive washing steps during the manufacture and formulation procedures. However, the presence of residual bound beads could be a concern, especially in the case of rapid manufacturing protocols. Although elements such as guide RNA and Cas9 proteins are expected to be degraded *in vivo*, new testing methods will be needed to assess their residual levels in products involving genome editing. For CAR-T cell products, especially autologous ones, the relevance of product-related impurities may be difficult to assess. For example, although residual blasts could have a clear impact on product quality and patient safety,^16^ the significance of residual untransduced T cells or NK cells is debatable. However, even if not forming part of the product specification for release, a broad characterization of the cellular content of each CAR-T batch is recommended and can be reviewed retrospectively in light of clinical responses.

#### Potency

For cell therapy products, potency assays are typically challenging to develop, as the determinants of therapy efficacy are complex and not fully understood. It is recommended that these assays are defined based on the expected mechanism of action, and, in the case of CAR-T cells, this generally includes assessment of cytotoxicity or cytokine secretion upon co-culture with cells expressing the targeted antigen. However, traditional methods, such as ^51^Cr release assays or evaluation of IFN-γ secretion by ELISA are complicated, labor intensive, and lack standardization.

Real-time cell analysis platforms, such as the impedance-based xCELLigence or the fluorescence-based Incucyte, can offer advantages over endpoint assays by providing information on killing kinetics, which is critical for identification of subtle differences among CAR-T products.^124^ Furthermore, microfluidics platforms are currently being developed and could represent an important step toward full automation for cytotoxicity analysis, allowing evaluation of multiple targets or even 3D tumor models in a single chip.^125^^,^^126^

Cytotoxicity data is often associated with additional assays, such as extended phenotyping for exhaustion and memory markers, or cytokine secretion profile, to provide a more comprehensive characterization of CAR-T products. High-throughput single-cell analysis platforms are useful for a systematic evaluation of the features of engineered CAR T cells and provide invaluable information on how they can be improved. Examples are RNA-seq platforms for single-cell transcriptome profiling^127^^,^^128^ and the IsoPlexis single-cell secretomics platform.^38^^,^^74^^,^^129^ As clinical data emerge, the variety of factors influencing *in vivo* efficacy are being explored by multiple groups and will provide additional insights on the critical quality attributes that determine product potency. This will likely inform the requirements of release-testing assays and help define potency criteria for CAR-T batches.

## Set up of phase 1 trials and challenges for academic teams

As a general rule, the path for CAR-T cell therapy development starts with academic discoveries, later spun out into start-ups or licensed to pharmaceutical companies. The importance of academic-industry collaborations in the delivery of the CAR-T products licensed to date is clear, but academic centers remain the primary players in the development of new modalities of these therapies.^130^ The academic/hospital setting has the advantage of a close proximity between researchers and patients, favoring translational research, and many of these centers already have licensed GMP facilities, conveniently located for production of autologous therapies.

With regard to the regulatory framework, a phase-appropriate approach is generally adopted by most agencies, including the EMA and FDA, when assessing the requirements for manufacture of ATMPs, including CAR-T cell therapies for use in clinical trials. For early-stage development, safety is a primary focus, but it is generally understood that other quality attributes may not be fully characterized. Encouraging innovation requires that regulatory requirements are adjusted to match the reality of academic centers and, in line with this, the EMA recently set up a pilot aiming to support academic and non-profit organizations on the translation of basic research developments into medicines for unmet needs. The program offers enhanced regulatory support, and participants benefit from available regulatory flexibilities and development support measures, such as fee reductions and waivers and details can be found in the EMA website at https://www.ema.europa.eu/en/news/ema-pilot-offers-enhancedsupport-academic-and-non-profit-developers-advanced-therapy-medicinal-products.

In our experience, an effective bench-to-bedside workflow (Figure 2) benefits from a close collaboration between research teams, manufacturers, and regulators from the earliest stages of development. Implementation of a robust manufacturing platform that can deliver consistent results, but is also flexible to adapt to new requirements and keep up with pre-clinical innovation, is also essential. In this setting, all-in-one automated devices have clear advantages, providing an easy-to-adapt starting point for new protocols, minimizing requirements with cleanroom, specialized workforce, and training. Academic manufacture needs to be dynamic, meeting the increased demand for new phase 1 trials, which typically recruit a limited number of patients, but require the delivery of highly innovative products, and often also be capable of scaling up manufacture for phase 2. A focus on product development, based on a QbD approach, is key and must take into account future requirements for later phases and scaling-out.

High costs and recruitment of trained manufacturing personnel are the main challenges currently faced by academic CAR-T manufacturers. Skills shortage is a critical problem in the Cell and Gene Therapy field as a whole but this is exacerbated in academic teams due to the high demand and competition from industry. On-the-job learning through an effective training program and collaboration with the University’s teaching activities to provide practical experience and internships for students enrolled in life science degrees can help narrow this gap, but the establishment of streamlined, standardized, and effective protocols, relying on automation, and with minimal hands-on requirements is essential. Overall, adoption of new manufacturing technologies and development of simple manufacturing processes will be critical in increasing availability, quality, and applicability of CAR-T cell therapies.

The setup of the academic CAR T program at University College London (UCL) illustrates many of the challenges and solutions discussed above for the delivery of a phase 1 clinical trial. A manufacturing platform based on the use of the CliniMACS Prodigy was implemented within the Center for Cell, Gene and Tissue Therapeutics (CCGTT), at the Royal Free Hospital, in London, and has been adapted for CAR-T cell production for 4 completed and 5 on-going phase 1 studies, with 5 more currently in implementation. These trials have recruitment cohorts of 12–40 patients and funding sources are varied, ranging from government bodies, charities, and non-profit organizations, as well as partnerships with the biotechnology industry. With a focus on the improvement of manufacturing processes and development of new clinical products, and benefiting from the close links with hospital sites and research scientists, delivery of over 100 CAR-T product batches with diverse requisites was facilitated by the use of a standardized protocol that is used as a starting point, but is also adaptable for a diverse range of requirements, such as autologous or allogeneic use, different cultivation conditions and different kinds of viral vectors (lentiviral/retroviral and concentrated/unconcentrated). Because the manufacturing science team consists of a small group of six to ten scientists, responsible for all stages of process development, assay development, preparation of product dossiers for regulatory submission, clinical manufacturing, QC testing, quality assurance, and logistics of all CAR-T batches and working in a multiuser facility where space is limited and disputed, automation and reduction of hands-on time are essential, as is the support of a core CCGTT team for facility management, quality management, and regulatory guidance. The clinical development pipeline adopted by the UCL program is described in Figure 2.

## Conclusions

The CAR-T cell manufacturing landscape is rapidly evolving. Automation currently emerges as a cornerstone, streamlining processes and enhancing scalability while mitigating the challenges posed by skills shortage and lack of consistency. Efforts to improve clinical outcomes rely on the robust characterization of CAR-T products, and will include not only engineering advancements but also improved manufacturing strategies to overcome terminal differentiation. Rapid manufacturing protocols, epitomized by next-day methodologies, not only tackle capacity limitations but are also expected to have an important impact on product efficacy. However, quality control remains a bottleneck to be addressed for further reduction of vein-to-vein times. Furthermore, the use of genome-editing technologies, notably CRISPR-Cas9, reshapes the therapeutic landscape, offering unparalleled precision in CAR-T cell engineering, but are associated with challenges such as genotoxicity and extensive requirements in safety evaluation and quality control implementation.

Academic teams play a crucial role in driving innovation and are essential for translating fundamental CAR-T research into clinical applications. Regulatory frameworks must adapt to the specific requirements of early-stage clinical trials, ensuring safety while creating an environment that supports innovation. As the field progresses, the alignment of technological advancements in manufacturing and quality control, associated with regulatory flexibility, can offer solutions to many of the current bottlenecks, accelerating the development and increasing availability of CAR-T cell products to a growing range of indications.
